# The Impacts of Drought on the Health and Demography of Eastern Grey Kangaroos

**DOI:** 10.3390/ani12030256

**Published:** 2022-01-21

**Authors:** Loic Quentin Juillard, Daniel Ramp

**Affiliations:** Centre for Compassionate Conservation, University of Technology Sydney, Ultimo, Sydney, NSW 2007, Australia; Daniel.Ramp@uts.edu.au

**Keywords:** eastern grey kangaroo, body condition, demography, drought, climate change, SPEI, NDVI, parasites

## Abstract

**Simple Summary:**

Eastern grey kangaroos, like most wildlife, are facing an increasingly uncertain future under rapid climate change. How individuals and populations cope with extreme climatic events will influence their capacity to adapt and persist. Here, we analyzed how drought impacted eastern grey kangaroo populations by focusing on their body condition, demography, activity rates at water points, and the likelihood of parasitic infections. We found that body condition was lower as environmental conditions became more extreme and that fewer males in the population were observed. The proportion of juveniles within the population increased as more favorable conditions returned. Kangaroos with poor body conditions were more likely to become hosts to ticks, while higher parasite egg burdens in scats occurred in autumn. Our study has shown that the impacts eastern grey kangaroos face during climatic events such as drought can be severe and may have long-term consequences.

**Abstract:**

Extreme climatic events such as droughts and floods are expected to become more intense and severe under climate change, especially in the southern and eastern parts of Australia. We aimed to quantify the relationship between body condition scores (BCS), demography, activity rate, and parasitic infections of eastern grey kangaroos on a large conservation property under different climate extremes by employing camera traps established at artificial water points (AWPs). The survey period included a severe drought, broken by a significant flooding event. Climatic and environmental conditions were documented using remotely sensed indices of moisture availability and vegetation productivity. These conditions were found to affect all health and population parameters measured. BCS, juvenile proportions, and sex ratios were most correlated with 6-month lags in climatic conditions, while the activity rate of kangaroos at AWPs was most correlated with vegetation productivity. Ticks were mostly found on individuals with a poorer BCS, while the concentration of parasitic eggs in feces was higher in autumn than in spring. Our study offers a glimpse into some of the environmental drivers of eastern grey kangaroo populations and their health, information that may become increasingly important in today’s climate. It further emphasizes the importance of this knowledge for wildlife conservation efforts appropriate to managing the impact of climate change alongside other threats.

## 1. Introduction

The global climate is changing at an unprecedented rate, leading to an increase in the frequency and intensity of extreme climatic events and posing significant threat to many wildlife species [1]. Natural phenomena such as droughts and floods are expected to become more intense and frequent as the world warms above 1.5 °C pre-industrial levels [2,3], due to an intensification of El Niño and La Niña events in many parts of the world [4]. On top of this, climate change is occurring in a world where wildlife are already facing mounting pressure from a range of anthropogenic activities, including habitat destruction and fragmentation, pollution, pathogens, and overexploitation [5]. The persistence of individuals and populations may hinge on their ability to adapt to extreme weather events caused by a changing climate [6], particularly as environments increase in aridity. The capacity for adaptation may disfavor species of temperate or mesic regions rather than species that are already arid-adapted. This may be especially noticeable for species in temperate regions with narrow climatic niches [7,8], but extreme events may also have an impact on the ranges of such species that currently extend into semi-arid regions [9]. Furthermore, the global climate is now changing so rapidly that many species simply do not have time to evolve resistance to it [10]. Understanding how wildlife are affected by events such as droughts and floods is therefore essential for addressing future issues in conservation [11].

While population abundance and ecological dynamics are often impacted by environmental change and climate, so are the health and welfare of wildlife [10]. Direct impacts during heatwaves can result in mass death events [12,13], but the long-term and gradual impacts of drought can lead to immunosuppression, disease susceptibility, and reproductive and developmental decline [6]. Reduced health and welfare can lead to a decline in the reproduction rate, skewing demography towards older populations as individuals become more likely to reach advanced life stages without successfully reproducing [14]. While extreme conditions are more likely to affect a population, conditions with lower intensity repeated over longer periods can also affect the body condition of individuals [15]. Body condition is known to be closely linked to the survival and reproduction rate of many species [16,17]. Poor body condition can impair the immune system, putting individuals at risk of pathogenic and parasitic infections [10]. Individuals with lower body conditions can also become more vulnerable to predation as they become too weak to flee from predators, or choose to trade costly behaviors such as vigilance for more vital ones such as feeding [16]. While parasite infections are often the cause of poorer body conditions, poor body condition can also make individuals more susceptible to parasite infection [18]. Furthermore, as body condition declines, individuals can suffer from defective immune systems as well as lower levels of maintenance and overall function, further increasing the likelihood of parasitic infection [18,19]. When food and water becomes scarce during drought, wildlife will often congregate at resource points in larger numbers than usual, increasing the risk of parasite transmission and outbreaks [20,21].

In Australia, drought is a pseudo-cyclic phenomenon that has occurred for millennia [22]. Under climate change, droughts in Australia are becoming more intense and lasting longer in southern and eastern parts of the country [3]. These temperate environments are likely to experience more frequent droughts and increasing aridity. While most anthropogenic structures exert pressure on wildlife, artificial water points (AWPs) may offer much-needed water [23,24]. Similar effects have been observed for water points dug by wildlife themselves [25]. Large herbivores, such as kangaroos, have been observed to utilize AWPs, particularly during hot periods when the vegetation is drier [26]. However, even though kangaroos may utilize AWPs to drink, there is no evidence that AWPs influence densities or assist with population growth [26,27]. While dingoes can suppress kangaroo populations through top-down regulation [28], in the absence of dingoes, kangaroo abundance and population dynamics are driven by primary productivity and the availability of food sources [29,30,31]. Despite this, access to water from AWPs and natural water sources is likely important for individuals in managing their health during drought [31].

Here, we aimed to detect changes in populations and health parameters of eastern grey kangaroos (*Macropus giganteus*) during two extreme weather events, namely, drought followed by flooding. To do so, we monitored body condition, demography, presence of parasites, and activity rates of eastern grey kangaroos at AWPs on a wildlife reserve in semi-arid south-western Queensland over a period of 18 months. Long-term changes in weather conditions were captured using the standardized precipitation evapotranspiration index (SPEI), which measures the driving effects of temperature on water demand [32], while shorter-term changes in the amount of live green vegetation were captured using the normalized difference vegetation index (NDVI) [33]. NDVI has previously been used to analyze the relationship between vegetation and wildlife performance [34].

We expected that the body condition of individuals would be lower during drier months (i.e., drought), and the use of AWPs would increase due to the lack of moisture in forage. We also predicted that higher body conditions would be observed in times of higher SPEI and NDVI, and that demographic shifts would occur as juvenile kangaroos become more frequent under favorable conditions due to increases in survival rates. The proportion of individuals with external parasites was expected to increase with the decreasing body condition and health of individuals.

## 2. Materials and Methods

### 2.1. Study Site

**Figure 1:**
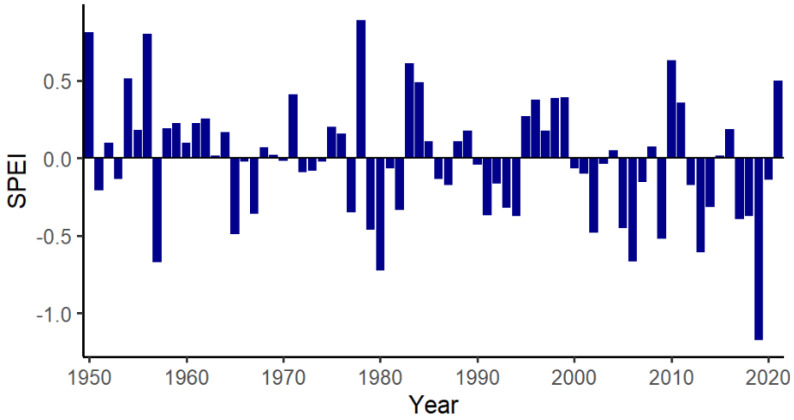
Yearly standardized precipitation evapotranspiration index (SPEI) average for the Mourachan Conservation Property from 1950 to 2021, showing 2019 as the driest year on record. SPEI values can be sorted into five classes: 1. non-drought (>−0.5), 2. mild (between −0.5 and −1), 3. moderate (between −1 and −1.5), 4. severe (between −1.5 and −2), and 5. extreme (<−2) [37].

The study was conducted on the 480 km^2^ Mourachan Conservation Property (MCP) in south-western Queensland, near the township of St George. This private semi-arid rangeland reserve, managed by Australia Zoo, is surrounded by cattle and sheep farms. Although a small number of cattle are run on one section of the property under wildlife friendly principles [35], the remainder is maintained as a conservation reserve where kangaroos and other wildlife are protected from persecution [36]. The MCP includes four macropod species: eastern grey kangaroos, red kangaroos (*Macropus rufus*), black wallabies (*Wallabia bicolor*), and red-necked wallabies (*Macropus rufogriseus*). Eastern grey kangaroos are distinguished from the only other large macropod, the red kangaroo, by body color and facial markings. Southern Queensland and most of Australia was in drought for the majority of 2019 due to El Niño, but weather conditions changed at the beginning of 2020, and the MCP was inundated with flood waters in late January 2020. Furthermore, 2019 was the driest year on record for the MCP (Figure 1).

### 2.2. Camera Traps

**Figure 2:**
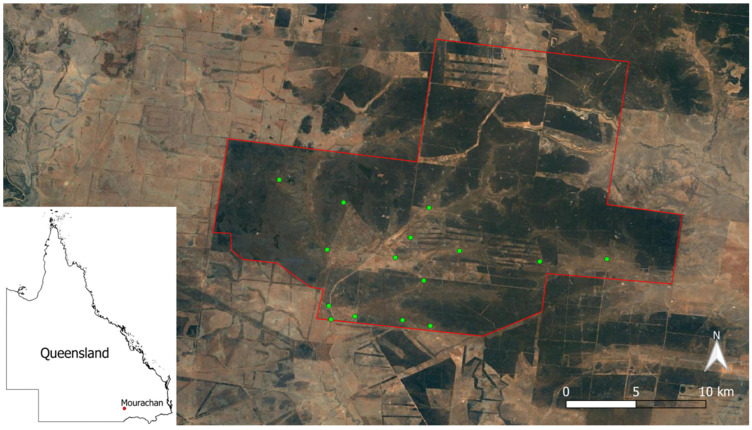
Location of the Mourachan Conservation Property within Queensland, showing the location of each artificial water point used within the property (green dots).

Camera traps (Strike Force HD Pro X, Browning, Morgan, UT, USA) were setup in November 2019 at 15 AWPs distributed across the property (Figure 2). At the time of installation, they were the only available sources of water on the reserve. A total of 40 cameras were used to capture motion-sensed photos from November 2019 to April 2021, leading to a total of 10,736 camera trap days. Some cameras were lost due to being completely submerged during flooding, while some temporal breaks in data capture occurred because of COVID-19 border restrictions that limited access to the site.

### 2.3. Body Condition Score and Demography

Eastern grey kangaroos were given a score between 1 and 5, depending on their visual body condition, using a similar method described by Johnston et al. [38]. A subjective body condition scoring system was developed, and example photos were collated for each score. Examples can be found in Appendix A Table A1. Scores were described as: 1 (emaciated), 2 (very thin), 3 (thin), 4 (average/healthy), and 5 (optimal). Images used to score BC were selected based on their quality, mainly relying on the full or most of the body being clearly visible. If more than one individual was present on an image, all kangaroos were scored. Only one image per event (pictures taken within 5 min of each other) was scored. A total of 3993 camera trap images were used. The demographic class of each scored kangaroo was also recorded using the classification references described by Austin and Ramp [39], using the following categories: pouch young, young-at-foot, sub-adult, small adult, medium adult, and large adult. The sex of each individual was determined using the description in Jarman, et al. [40].

### 2.4. Activity Rate

The activity rate of kangaroos was measured to analyze how often they use artificial water points, both during drought and under more favorable conditions. We recorded the total number of kangaroo events per month, defining events as a series of camera trap images captured within 5 min of the previous image, where one or more kangaroos were visible. The number of kangaroos visible on an image did not influence the number of events (i.e., images within 5 min of each other with more than one kangaroo were still counted as a single event). The activity rate here, therefore, does not represent the number of individuals within the population, only how often kangaroos were observed using the AWPs.

### 2.5. Parasitology

The presence/absence of parasitic ticks was recorded from camera trap images. The images used to analyze tick presence were the same images used to measure BCS due to their higher quality. Rather than analyzing the relationship between tick-infected individuals and environmental conditions, we focused on the relationship between the presence of ticks and the BCS of the kangaroos.

Kangaroo scat samples were used to estimate the average Fecal Egg Count (FEC) of the population during drought (2019) and post-flood (2021). Where possible, fresh scat samples were collected directly after observing foraging eastern grey kangaroos. When no kangaroos were present, samples were collected based on the size and shape of the scat [41]. We validated that the scats collected in November 2019 were from eastern grey kangaroos through genetic analysis [36]. Samples were collected from each AWP in November 2019 (spring) and stored at −20 °C. It should be noted that storage at −20 °C can lead to some loss of eggs in the scats due to biological degradation [42]. Samples were also collected in April 2021 (autumn). FECs were performed by mixing 3 g of a scat sample in 60 mL of saturated salt solution. Eggs were counted using a Whitlock Universal 4 chamber worm egg counting slide (J.A. Whitlock and Co, Eastwood, Australia), following the methods described by Gordon and Whitlock [43].

### 2.6. Statistical Analysis

General linear mixed models (GLMM) were used to analyze the effects of climatic conditions and primary productivity on the body condition, demography, and activity rate of the population. Models for body condition and activity rate used a Poisson distribution, while demography used a binomial distribution. SPEI values were used to represent long-term trends and were, therefore, used with lags of 3, 6, and 12 months to represent a quarter of a year, half of a year, and a full year. NDVI was used to represent more immediate and short-term changes in health and population parameters related to primary productivity and was, therefore, used with a monthly lag. SPEI time series was downloaded from the SPEI Global Drought Monitor using the MCP as a single grid cell [44]. We used a polygon of the MCP as a mask to select tiles and obtain monthly NDVI values from the MODIS Terra NDVI composites [45]. GLMMs (generalized linear mixed model) with binomial distribution were also used to determine whether the body condition of kangaroos influenced the likelihood of tick infection. A *t*-test was performed to compare the Fecal Egg Count from scats between November 2019 and April 2021.

All GLMMs were performed in R v4.1.1 (R Foundation for Statistical Computing, Vienna, Austria) [46] using the “glmer” function of the “lme4” package (Version 1.1-27.1) [47].

## 3. Results

### 3.1. BCS and Demography

**Figure 3:**
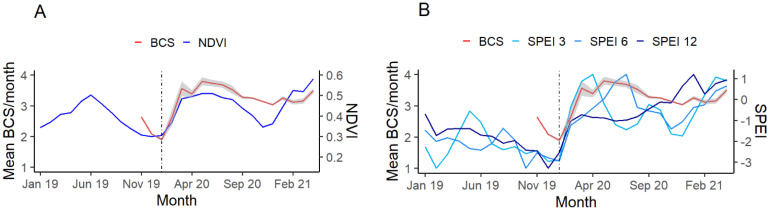
Temporal changes in the mean body condition score (BCS) of eastern grey kangaroos; environmental conditions represented by the Normalized Difference Vegetation Index (NDVI) (**A**); and standardized precipitation evapotranspiration index (SPEI) with a 3, 6, and 12-month lag (**B**). The black reference line represents the January 2020 floods and the end of the 2019 drought. The shaded confidence interval around BCS represents the standard error of the mean.

There was a strong positive relationship between SPEI and BCS, with the 3-month lag (SE = 0.011, z = 13.93, *p* = <0.001, and r = 0.6) and 12-month lag (SE = 0.009, z = 14.86, *p* = <0.001, and r = 0.57) showing similar patterns (Figure 3). However, a stronger relationship was observed between BCS and SPEI with a 6-month lag (SE = 0.013, z = 15.35, *p* = <0.001, and r = 0.87). Similarly, NDVI was also shown as being significantly correlated with BCS (SE = 0.156, z = 12.046, *p* = <0.001, r = 0.8) (Figure 3).

**Figure 4:**
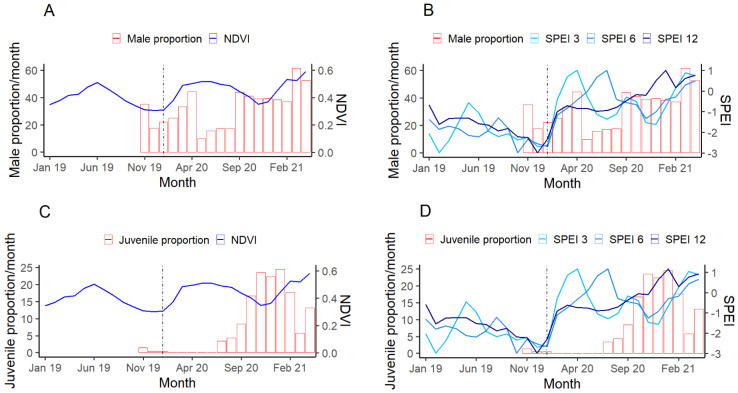
Temporal changes in the monthly proportion of male eastern grey kangaroos within the observed population compared to the normalized difference vegetation index (NDVI) (**A**); standardized precipitation evapotranspiration index (SPEI) with a 3, 6, and 12-month lag (**B**); and proportion of juvenile kangaroos compared to NDVI (**C**) and (SPEI) (**D**). The black reference line represents the January 2020 floods and the end of the 2019 drought.

SPEI with a 3-month lag (SE = 0.047, z = 8.612, *p* = <0.001, and r = 0.54) and a 12-month lag (SE = 0.037, z = 10.11, *p* = <0.001, and r = 0.62) were found to have the strongest effect on the sex ratio of the population, with fewer males observed using AWPs during drier conditions. SPEI values with a 6-month lag (SE = 0.051, z = 7.527, *p* = <0.001, and r = 0.14), and NDVI values (SE = 0.634, z = 5.643, *p* = <0.001, and r = 0.21) were found to have the weakest relationships with sex ratio. The ratio balanced out with the return of more favorable conditions (Figure 4A,B).

The monthly adult to juvenile ratio of the MCP population visiting AWPs had a relatively weak relationship with SPEI at 3-month lags (SE = 0.065, z = 7.896, *p* = <0.001, and r = 0.18), and NDVI (SE = 0.863, z = 5.48, *p* = <0.001, and r = 0.14). Stronger effects came from SPEI with longer term lags, as shown by values with a 6-month lag (SE = 0.079, z = 11.824, *p* = <0.001, and r = 0.23) and a 12-month lag (SE = 0.081, z = 14.767, *p* = <0.001, r = 0.31).

### 3.2. Activity Rate

**Figure 5:**
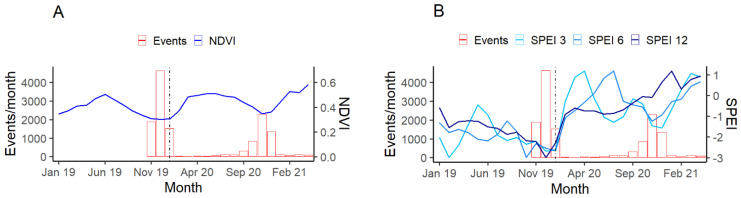
Temporal changes in the number of eastern grey kangaroo events per month compared to shifts in environmental conditions represented by the normalized difference vegetation index (NDVI) (**A**), and standardized precipitation evapotranspiration index (SPEI) with a 3, 6, and 12-month lag (**B**). The black reference line represents the January 2020 floods and the end of the 2019 drought.

Activity rates (average number of events per month) of eastern grey kangaroos significantly declined at AWPs as weather and vegetation indices increased after the drought broke (SPEI with a 3-month lag SE = 0.013, z = −72.00, *p* = <0.001, r = −0.72, a 6-month lag SE = 0.013, z = −71.286, *p* = <0.001, r = −0.75, a 12-month lag SE = 0.01, z = −63.667, *p* = <0.001, r = −0.59 and NDVI SE = 0.194, z = −69.01, *p* = <0.001, and r = −0.77). This shows that drier conditions increased the likelihood of kangaroos accessing AWPs (Figure 5A,B).

### 3.3. Parasitology

**Figure 6:**
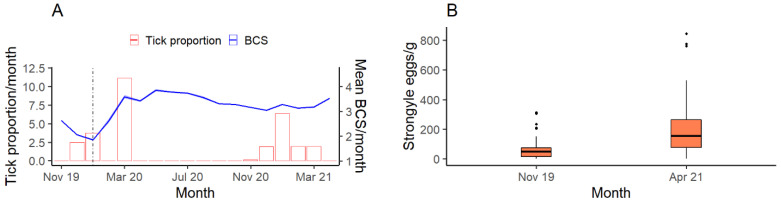
(**A**) Relationship between the proportion of kangaroos infected with ticks and their body condition according to a generalized linear mixed model (GLMM) using a Binomial distribution. The pale blue band represents a 95% confidence interval. (**B**) Number of Strongyle eggs per gram of kangaroo feces for samples collected in November 2019 during the drought (*n* = 82), and in April 2021 (*n* = 42). Outliers are represented by dots. BCS: body condition score.

The number of kangaroos infected by ticks increased as their body condition declined (SE = 0.181, z = −3.712, *p* < 0.001, r = −0.4). Ticks were more likely to be observed on individuals with poorer body conditions. Fecal egg counts for April 2021 (post-flood) were found to be significantly higher than November 2019 (drought) (df; degrees of freedom = 41.971, t = −3.644, *p* = 0.001) (Figure 6A,B). All eggs observed were identified as *Strongyle* eggs.

## 4. Discussion

We found that the body condition of eastern grey kangaroos was negatively correlated with environmental factors explained by climatic conditions, as measured by long-term trends in moisture availability (SPEI) and primary productivity (NDVI). As both SPEI and NDVI increased, leading to more favorable conditions, the body condition of kangaroos increased. We also found that the SPEI data with a 6-month lag had the strongest relationship with BCS. However, this does not suggest that NDVI values cannot be used to predict a relationship between BCS and primary productivity since a correlation of r = 0.8 was found between NDVI and BCS. In other species, such as the roe deer (*Capreolus capreolus*) and red deer (*Cervus elaphus*), NDVI was found to be a strong predictor of body mass [34,48]. NDVI has also been shown to influence the body condition and reproductive timing of the African buffalo (*Syncerus caffer*) and African elephant (*Loxodonta africana*) [49]. Poor body condition of animals during drought is thought to be one of the major causes of deaths [50]. For example, Knight [50] found that 90% of fresh carcasses (of four species of ungulates) found during droughts in the southern Kalahari had poor body conditions. In Australia, the body condition of the southern hairy-nosed wombat (*Lasiorhinus latifrons*) is closely related to food availability and quality [51]. Kangaroos often choose greener, more nutritious vegetation, resulting in high mortality rates during drought when such resources are not available [52]. Poor body condition often leads to adverse health effects such as lower immune responses due to impaired immune systems, increasing infection likelihood while lowering resistance [10]. This can also impair nutrient uptake and lead to poor nutritional status [53]. Nutritional and hydrological stresses, such as drought, are known to affect other processes such as growth and reproduction, over and above increased mortality during these events [54].

The sex ratio of eastern grey kangaroos visiting AWPs at Mourachan shifted during drier months, with fewer males being observed when environmental conditions were poorer (Figure 4A,B). However, the increase in males observed post flood did not result from juvenile male births but from the adult individuals. This suggests that some of the males were alive during the drought but simply did not use the water points as much as the females did. Previous studies on the mortality of kangaroos during droughts have reported that males had a higher mortality rate than females [55,56], which manifested itself through all age classes and is thought to have been caused by the differences in energetic costs, body size, and mobility between the sexes [55]. As the sex ratio of eastern grey kangaroos in our study returned quickly to parity and was not driven by recruitment, it is likely that behavioral decisions may explain this trend.

The proportion of juvenile kangaroos within the population increased as environmental conditions improved post drought and exhibited a stronger relationship with longer-term data (SPEI 6 and 12) than short-term (Figure 4D). Reproduction in kangaroos is driven by biological and ecological constraints. Gestation period in this species is approximately 36 days [57] and is influenced by mother health and climatic conditions. Reproduction is more successful when females have a high BCS [52]. Juvenile kangaroos represented below 2% of observations at the height of the drought and were absent until July 2020, four months after the floods (Figure 1). For many mammal species, the energy requirements of reproduction can lead to a reduction in female body mass [58], requiring females to feed more to compensate for the energy allocated towards reproducing [59]. Further, eastern grey kangaroos are seasonal breeders and embryonic diapause is rare [60], meaning that response to favorable conditions may be delayed as individuals take time to improve body condition and synchronize with typical reproductive cycles. Under favorable conditions at Mourachan that began in autumn 2020, the proportion of juveniles gradually increased and peaked at the beginning of summer 2020/2021, where they comprised around 25% of observations. However, despite generally good conditions in the summer of 2020/2021, observations of juveniles declined to around 5% as summer progressed, possibly due to juvenile mortality as they are more vulnerable to higher temperatures and a lack of moisture in vegetation than adults [61,62]. In previous studies, juveniles and older kangaroos have been shown to be the first to die during droughts [56].

For arid-adapted macropod species, such as the red kangaroo (*Macropus rufus*), rearing juveniles can cost the mother as much as 50% of her own daily energy requirement for maintenance near the end of the juvenile’s pouch stage [52]. If environmental conditions are favorable and the condition of mothers is high, up to 85% of pouch young can make it to the weaning stage [52,63]. During drought, the costs of caring for young can often surpass the daily energy intake of the mother, leading to higher rates of juvenile mortality [64]. Red kangaroo mothers have the adaptive advantage of suspending embryonic development (embryonic diapause) while environmental conditions are poor [52], a trait that has evolved to promote survival under arid conditions [60]. Without this advantage, grey kangaroos are less resilient to drought conditions. It remains to be seen whether eastern grey kangaroos under increasing aridification can utilize diapause more commonly than current evidence suggests. What is apparent, however, is that red kangaroos may be physiologically better suited to survive droughts than eastern grey kangaroos [65].

We also found that environmental conditions affect the activity rate of kangaroos at artificial water points, with more events occurring during drier months when less water and live green vegetation was available across Mourachan (Figure 5). Eastern grey kangaroos access water points to drink before returning to a more favorable grazing site [26], while food availability is a greater driver of dispersion and density. Kangaroos living in rangelands often drink at AWPs, especially during summer when vegetation availability and moisture content are low. As growing vegetation became available after the flood, we found that kangaroos spread out and were less reliant on AWPs. Wildlife activity in semi-arid and arid ecosystems is often focused around sources of water, but while AWPs were originally thought to increase the abundance and density of kangaroos [66], it has now been accepted that AWPs do not influence their density [24]. In accordance with this, we suggest that changes in activity rates at AWPs at Mourachan were likely a response of changing physiological requirements due to the lack of moisture in vegetation, a circumstance that may become more challenging as droughts are predicted to become more frequent and intense.

Tick infections were increasingly prevalent on eastern grey kangaroos with lower BCS (Figure 6A). The presence of ticks may contribute to declines in health, depending on the type of tick and the location on the body, but poor health may also make kangaroos more susceptible to ticks. One explanation for the latter relationship is that the grooming rate of the unhealthy kangaroos can decline. Grooming costs can include loss of saliva, leading to a loss of water, as well as a reduction in vigilance rates [67]. When in poor physical condition, various species will often reduce behaviors such as grooming to focus their remaining energy on more vital activities such as foraging [67]. This gives parasites, such as ticks, the opportunity to attach themselves to hosts without the risk of being removed, increasing the likelihood of infection [18]. Eastern grey kangaroo mothers often groom their young [68], and we did not detect any ticks on any juveniles we observed, which could be due to the mother removing the ticks while grooming the young. Although we could not identify ticks to species level in our study, three species of ticks in Australia have been confirmed as causing paralysis in species such as kangaroos [69]. The prevalence of ticks on kangaroos, and their impact on the condition, health, and resilience to a changing climate, remains unknown.

The fecal egg count data revealed that the number of eggs per gram of scat was significantly higher in autumn than in spring, suggesting cooler, wetter conditions could be more favorable to parasites such as strongyles (Figure 6B). Kangaroos naturally carry a variety of gastrointestinal parasites [70]; however, strongylid nematodes are the most abundant in eastern grey kangaroos [70,71]. Juvenile kangaroos are the most at-risk, especially from trichostrongylid nematodes such as *Globocephaloides trifidospicularis*. Parasite infections in eastern grey kangaroos are more prevalent in winter during wetter conditions [72]. While the post-flood samples were not collected in winter, the flood would have created conditions more suited to parasites than the drought [73]. Floods can also bring in more parasites such as *Fasciola hepatica*, which use freshwater snails as intermediate hosts [73]. While we cannot attribute differences in parasite load to drought/flood cycles because we were unable to replicate sampling to accommodate seasonal or annual trends, we nevertheless note the potential for changes in patterns of parasite prevalence to impact on the disease burden of kangaroos, which may be heightened by increasing extreme conditions.

## 5. Conclusions

The impacts on the health and demography of eastern grey kangaroos identified in this study are only some of the effects caused by environmental events such as droughts. While droughts have been a part of Australia’s climate for thousands of years [22], predictions of increasing prevalence are concerning. Droughts in the southern and eastern regions of the country are expected to last longer, while reaching more extreme intensities due to the intensification of El Niño and La Niña events [4]. Much of the wildlife in Australia has evolved with droughts; however, rapid changes in global climate may reduce the resistance of many species to droughts [10]. Species such as kangaroos may suffer from poorer body conditions, leading to an increased risk of infections by parasites [18]. Lower body condition could also lead to lower birth rates due to the poor fitness of females, while juveniles may have higher mortality rates [52]. These challenges, along with current threats from human activities [61,74], may contribute to further declines in kangaroo populations. From a global perspective, climate change causes new challenges for wildlife conservation. It is therefore essential to understand how wildlife is affected by climate change to address this issue [11]. To improve our knowledge of the impacts of climate change on eastern grey kangaroos, longer term studies located in different ecosystems would be required to cover more populations. While our study showed a contrast between drought and flood, it only covered one drought/flood cycle; a longer project would, therefore, allow for the comparison of multiple cycles and could further clarify patterns we have found. Studies covering a longer period would also allow for the collection of data on parameters such as the growth rate and survival of juvenile kangaroos, while also enabling yearly comparisons of the reproduction rate, BCS, and demography of populations.

## Data Availability

Data sharing is not applicable to this article.

## Appendix A

**Table A1:** Descriptions and examples of the body condition scores used in the study.

BCS	Description	Example Camera Trap Photos
5 (Muscular)	Thick tail with a large base. Moderate fat cover on body. Muscle definition highly visible across most of the body, especially biceps and forearms, chest, and legs.	
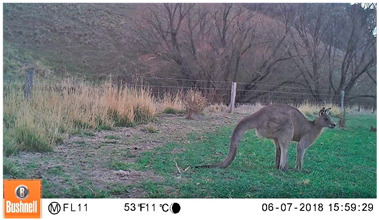
4 (Optimal)	Thick base of tail. Hips covered by fatty tissue. Ribs not visible. Hips well covered with no visibly sunken area or bones protruding. Muscle mass well developed with generally minimal fat cover on the body.	
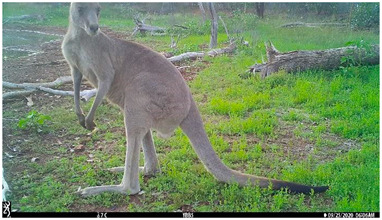
3 (Thin)	Tail appears thin but no caudal vertebrae are visible. Some ribs may be visible, but the rib cage area is mostly covered. Hips are generally well covered, and muscle mass mostly visible. There is no visible fat cover.	
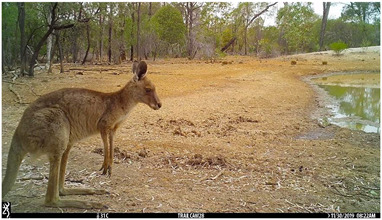
2 (Very thin)	Most caudal vertebrae are visible, giving the tail a bony appearance. Tail base is skinny. Hip region is mostly without fat or muscle tissue cover, creating sunken appearance above the femur (as seen in the visual example). Most ribs are visible (and are easily distinguishable on individuals with shorter fur as pictured here).	
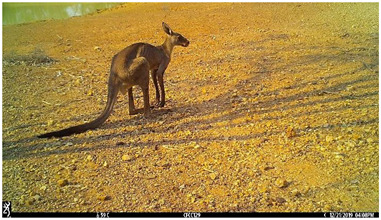
1 (Emaciated)	Caudal vertebrae are visible, giving the tail a bony appearance. Base of tail appears skinny. Hip region is not covered by fatty tissue and is sunken in appearance. All ribs are visible (it may be difficult to distinguish if the individual has longer fur as pictured here).	
